# Introduction to Special Issue: Genomic Analysis of Common Disease

**DOI:** 10.3390/cimb47020112

**Published:** 2025-02-10

**Authors:** Golder N. Wilson

**Affiliations:** 1 Department of Pediatrics, Texas Tech University Health Sciences Center, Lubbock, TX 79430, USA; golderwilson@gmail.com; 2 KinderGenome Genetics Private Practice, 5347 W Mockingbird, Dallas, TX 75209, USA

The development of NextGen or massive parallel sequencing [1] has transformed genetic analysis and its ability to sequence a billion DNA nucleotides an hour, allowing for rapid scanning of genomes and populations for medically significant variations [2,3]. This Special Issue shows how NextGen sequencing, analogous to reading all pages of a textbook at once, can move beyond rare genetic diseases associated with single-nucleotide changes [4] to analyze common conditions influenced by multiple genes and the interior/exterior organismal milieu [5].

Just as epidemiologic studies reveal much more about sickle cell anemia than its uniform beta-globin mutation [4], the studies of Fylaktou et al. [6] on 21-hydroxylase deficiency, Evangelidis et al. [7] on hemophilia, and Balinisteanu et al. [8] on acromegaly broaden the view of single-gene disorders when a range of contributing mutations and genes are examined. Adding to this theme are articles on cancers that occasionally have germline but always somatic gene change. For example, Baek et al. [9] analyze the transcript patterns of pre- and post-transcript cervical cancers, Cassoli et al. [10] focus on the multiple germ-line variants of Her2-low breast cancers, Tan et al.’s [11] study echoes the epigenetic focus of Evangelidis et al. [7] by showing how this process might translate cell-free DNA change into cancer biomarkers, and Alhatim et al. [12] question how the presence of carcinomas might affect DNA testing for autosomal traits and DNA fingerprinting for identity.

Articles examining particular examples of common disease processes include Cao et al. [13], which again considers epigenetic changes as they might correlate with body fat phenotypes; Minelli et al. [14], which uses the technique of microarray analysis to describe a 1q21.1 microduplication and emphasize the variable phenotype associated with even the smallest of genomic imbalances; Treccani et al. [15], which looks at the multifactorial contribution to a form of eosinophilic granulomatosis; Guarienti et al. [16], which examines whether variations in the angiotensin-converting enzyme ACE and the COVID-19 receptor *ACE2* genes predict outcomes of viral infection; and our article [17], which focuses on the many genes contributing to the most common connective tissue dysplasia called Ehlers–Danlos syndrome.

Unifying these articles are words from the title of Evangelidis et al. [7]: from bench to bedside. Although the ability to relate biochemically disruptive mutations to disease by unbiased genome scanning [2,3] has medical advantages over the polymorphism associations of low-cost DNA testing [18] or whole-genome association studies [19], the involvement of clinicians in the qualification of DNA variant significance [5] is needed to allay insurance and physician skepticism [20] about the value of gene panel and whole-exome sequencing. Collaborations of experienced subspecialists with attuned molecular biologists, as exemplified here, promise precision DNA medicine that goes beyond coincidence or correlation to gene causation, prevention, and therapy [21].
